# Chitosan Interaction with Iron from Yoghurt Using an *In Vitro* Digestive Model: Comparative Study with Plant Dietary Fibers

**DOI:** 10.3390/ijms12074647

**Published:** 2011-07-19

**Authors:** Marina Dello Staffolo, Miriam Martino, Alicia Bevilacqua, Mirta Montero, María Susana Rodríguez, Liliana Albertengo

**Affiliations:** 1 Centro de Investigación y Desarrollo en Criotecnología de Alimentos (CIDCA), CONICET–CCT La Plata, Fac. Cs. Exactas, Universidad Nacional de La Plata, La Plata, 47 y 116, 1900, Argentina; E-Mails: mmartino@ing.unlp.edu.ar (M.M.); aebevila@ing.unlp.edu.ar (A.B.); 2 Instituto de Química del Sur (INQUISUR), CONICET–CCT Bahía Blanca, Departamento de Química, Universidad Nacional del Sur, Av. Alem 1253, Bahía Blanca, 8000, Argentina; E-Mails: mmontero@criba.edu.ar (M.M.); mrodri@uns.edu.ar (M.S.R.); alberten@criba.edu.ar (L.A.)

**Keywords:** chitosan, iron, yoghurt, gastrointestinal simulation, plant fibers

## Abstract

The objective of this work was to investigate the interaction of chitosan with iron from yoghurt by an *in vitro* gastrointestinal tract model. Taking into account that chitosan is a polysaccharide included in fiber definition by Codex Alimentarius; chitosan behavior was studied and compared with different plant fiber (wheat, bamboo, apple, psyllium and inulin) behaviors, in the same *in vitro* conditions. Ferrous sulfate was added to yoghurts with each type of fiber. The gastric environment was simulated with HCl (pH 1.0–2.0). The duodenal environment was simulated with NaHCO_3_ (pH 6.8–7.2) and a dialysis tubing cellulose membrane. Results showed that chitosan had the highest iron retention percentages (53.2% at 30 min; 56.8% at 60 min) interacting in a more pronounced manner with iron than the plant fibers used in this work.

## 1. Introduction

Chitin is an amino-polysaccharide containing β-(1,4)-linkages as is present in cellulose. It is a constituent of exoskeletons of arthropods and cell walls of most fungi. Chitosan, the principal chitin derivative, is a heteropolysaccharide consisting of linear β-1,4-linked GlcN and GlcNAc units. This carbohydrate polymer is resistant to hydrolysis by human endogenous enzymes and is biocompatible and biodegradable. Moreover, chitosan has no toxicity in animal organs [1–3].

The Codex Alimentarius Commission adopted a new definition of fiber in July 2009, designed to harmonize the use of the term around the world. It describes fiber as elements not hydrolyzed by endogenous enzymes in the small intestine (indigestibility) as well as having physiological effects beneficial to health. Dietary fibers are carbohydrate polymers with ten or more monomeric units and belonging to one of three categories of carbohydrates polymers: edible carbohydrate polymers naturally occurring in food, carbohydrate polymers which have been obtained from raw food material by physical, enzymatic, or chemical means (chitosan is included in this group); and synthetic carbohydrate polymers [4–6]. Because chitosan is an animal-origin carbohydrate polymer, which is included in the definition, it is being used as a new source of dietary fiber helping to meet consumer requirements all over the world that are increasingly interested in products high in dietary fiber [7,8].

Minerals and trace elements have gained increasing interest in nutrition fields. Iron (Fe) deficiency is a leading nutritional concern worldwide, affecting 20–50% of the world’s population [9]. There is an unequivocal need for predicting absorption of dietary iron [10]. The aim of most of the investigations in this field is to make evident that fiber may be an important determinant of the utilization of minerals in the diet. Much research has been done to better understand the physicochemical interactions between dietary fiber and minerals in the past decades [11–13]. Several of these investigations have applied *in vitro* digestive models to study iron absorption in foods [14–16]. Some studies have been done with cereal foods because of the known capacity of phytate to bind minerals [17,18]. However, few works have been done to study iron absorption from fermented milk products [19].

Yoghurt is one of the best known food products that may contain probiotics and is currently increasing supplementation with prebiotics, a type of fiber that stimulates the growth of specific bacteria in the gut [20]. Synbiotic is a new concept to describe this kind of product and is popular among dairy manufactures in Europe [21]. In addition, yoghurt is a suitable food for iron fortification because fermentation markedly increases iron dialyzability and ferrous sulfate is known as having the highest bioavailability [22].

Considering the fact that chitosan is a new ingredient widely applied in foods and that there is a need of recognizing enhancers and inhibitors of iron absorption, the current work was designed to study the interaction of chitosan with iron when it was added to yoghurt as a food model. This interaction, evaluated as iron percentage retention, was compared with the behavior of different plant fibers: wheat, bamboo, apple, Psyllium and inulin. To chemically characterize the fibers used in this work, initial measurements of total solubility, insolubility, NDF (Neutral Detergent Fiber), ADF (Acid Detergent Fiber), cellulose, hemicellulose and lignin were taken. Then an *in vitro* digestive model was employed to quantify iron retention percentages of chitosan and different plant fibers.

## 2. Results and Discussion

Trends in the area of food and nutrition include the introduction of new ingredients, like chitosan, to make functional foods. Consequently, there is a continuous need for predicting the interactions between chitosan and mineral nutrients, like iron.

In a previous work, we studied sensory and rheological properties of yoghurts fortified with the same plant fibers as we used in the present article (apple, bamboo, inulin and wheat) [23]. Moreover, we evaluated the interaction of chitosan and oil, using an *in vitro* chemical experimental model of the human digestive tract (gastric and duodenal environment) [24]. In another work we demonstrated that when chitosan is added to a food like yoghurt, both glucose and calcium availabilities are decreased and this effect is more pronounced than that produced by plant fibers. We also demonstrated using the Association of Official Analytical Chemists (AOAC) method, that fiber content in chitosan samples was higher than 92% [25]. All these results allow us to confirm that chitosan behaves as a dietary fiber. Based on the premise that yoghurt is a good vehicle for both viable probiotics and prebiotics, and it is a suitable food for iron fortification, we studied chitosan interaction with iron from yoghurt as a food model.

### 2.1. Characterization of Fibers

The dietary fibers used in this study have different water solubility characteristics: inulin is a soluble fiber, bamboo and wheat are insoluble fibers, apple is partially insoluble fiber, and psyllium forms a viscous dispersion at concentrations below 1% and a clear gelatinous mass at 2%. Chitosan is a fiber of a different origin, *i.e.* from animal source and is soluble in an acidic medium and flocculates in an alkaline medium. We used these fibers because they present different physicochemical behaviors that have been described in literature [2,26]. The commercial fiber compositions used in this study, regarding total, soluble and insoluble fractions, are shown in Table 1. Analysis for dietary fiber using the AOAC method 991.43 showed that wheat and bamboo have high amounts of insoluble fraction.

Inulin presents only soluble fraction in concordance with suppliers. Psyllium and apple have both soluble and insoluble fractions. The total dietary fiber content is 45.2% for psyllium, which is an acceptable value, taking into account that the supplier declared a 49.15% of Plantago ovata seed husk in Metamucil preparation and Van Craeyveld *et al*. [28] reported 3.4% (dm) ash and 7.1% (dm) protein contents for Plantago ovata seed husks. The total dietary fiber content is 58.1% for apple, which is about 10–14% higher than the values reported by Sudha *et al*. [29], however, this value was in accordance with suppliers.

The chitosan used in this study has 98% of insoluble fraction and no detectable soluble fraction. Furthermore the characteristics of this biopolymer are a deacetylation degree of 89%, a viscosity of 120 mPa.s, a 6.7 g% moisture and a 0.67 g% ash content.

Plant fiber characterizations were completed with the study of Acid Detergent Fiber (ADF) and Neutral Detergent Fiber (NDF), lignin, cellulose and hemicellulose contents (Table 2). Apple presents the highest lignin content. Wheat fiber mainly has cellulose. Bamboo has proportional amounts of cellulose and hemicellulose, but compared with other fibers, has the highest hemicellulose content. These results are in accordance with their plant fiber origins and previous works [28–30]. Frutafit-Inulin was not analyzed because its composition was ≥85.5% (w/w) of inulin, ≤9.5% of mono and disaccharides, ≤0.1% of ash with degree of polymerization ≥9 according to suppliers. Chitosan was not analyzed either, because of its animal origin.

Scientists who deal with animal nutrition usually use Van Soest’s method to analyze feed. Scientists working on human nutrition use methods of the AOAC, because of their interest in soluble fiber. It is known that soluble fiber plays an important role in human health and the food industry. However, it could be useful in human nutrition to know the composition of insoluble fiber, as it is possible that insoluble fibers do not all have the same effect on human health. The NDF and insoluble fiber methods were applied to the same samples. Insoluble fiber includes hemicellulose, cellulose, lignin, cutin, suberin, chitin, chitosan, waxes and resistant starch. NDF includes hemicellulose, cellulose and lignin. Escarnot *et al*. [32] studied three wheat varieties and four spelt genotypes. They analyzed three milling fractions from those grains for insoluble and soluble fiber contents, lignin, hemicellulose and cellulose. They found a very high correlation (r^2^ = 0.99) between the two methods, showing that NDF and insoluble fiber methods cover the same types of fiber. For insoluble fiber analysis, the NDF method is faster and more thorough.

### 2.2. Digestive Chemical Model and Iron Retention Percentages

*In vitro* digestion approaches have obvious limitations, but they have been employed as a useful tool, particularly for screening samples before elaborating *in vivo* and expensive human studies. In the present study, the introduction of cellulose dialysis tubes in the digestive chemical experimental model is utilized to study iron retention by the fibers tested. The use of a membrane dialysis tube reproduces, in the laboratory, the duodenum wall and according to Miret *et al*. [33] its utilization, is presumably a significant factor that determines iron absorption. This type of model allows current research needs for fast, cheap and efficient experimental procedures. Digestive enzymes were not utilized in this model because they do not hydrolyze fibers. The importance of duodenal simulation in this study is because most dietary iron is absorbed in the duodenum.

In this work, yoghurts with each type of fiber are added with 0.8% (w/w) of ferrous sulfate. In yoghurt, caseins are modified as a consequence of its production process. Bioactive peptides are formed from caseins during the elaboration of milk products (cheese, yoghurt) under the action of endogenous enzymes of milk (plasmin, cathepsin, among others) or of microorganisms [34].These peptidic fragments that are already present in yoghurt, could fix iron according to Bouhallab and Bouglé [34]. Then, these complex matrixes (yoghurts with each type of fiber and iron) are subjected to the gastrointestinal simulation. A control yoghurt with ferrous sulfate without fiber was also subjected to the digestive simulation and considered to be 0% iron retention (100% iron dialyzated) to calculate iron retention percentages for each fiber. With this control yoghurt, we could consider the interaction of iron with casein peptidic fragments.

**Figure 1:**
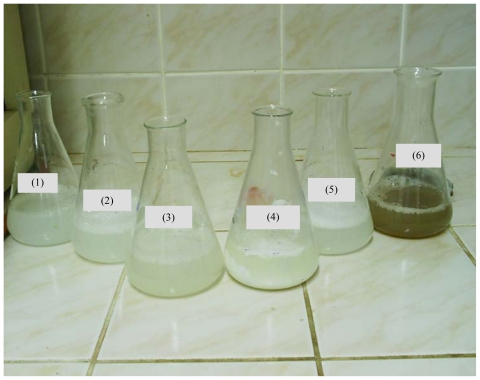
Simulation digestive mediums of different fibers before dialysis (37 °C, pH 6.8–7.2 with 0.2 M NaHCO_3_ and stirring speed 300 rpm to reproduce the chemical duodenal environment). (**1**) Inulin, (**2**) Bamboo, (**3**) Psyllium, (**4**) Chitosan, (**5**) Wheat, (**6**) Apple.

**Figure 2:**
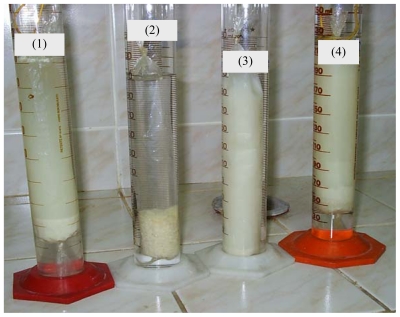
Different fiber behaviors in the dialysis step of digestive simulation. (**1**) Yoghurt without fiber, (**2**) Chitosan, (**3**) Psyllium, (**4**) Wheat.

Simulation of gastrointestinal environment of different yoghurts can be observed in Figure 1 (before dialysis) and Figure 2 (during dialysis). Changes in pH during gastrointestinal simulation produces different behaviors depending on the type of fiber employed. Apple fiber shows brownish color (Figure 1), probably due to the content of phenolics compounds [35]. In Figure 2 it can be seen that Psyllium fiber gives a viscous dispersion [36,37]. Due to changing pH values in the digestive tract, Chitosan precipitates while passing through the first portion of the small intestine, forming flocculus. Chitosan a positively charged polysaccharide that is insoluble in neutral and alkaline pH. It is only soluble in acidic pH because below pH 6.5 (pK_a_ = 6.5), the amine groups of chitosan are positively charged. When it is solubilized in dilute acid, chitosan has a linear structure [38]. At pH > 6.5, the polymer loses its charges from the amine groups and therefore becomes insoluble in water and precipitate forming flocculus.

When chitosan is added to yoghurt, one might think that chitosan would remain soluble. However, yoghurt contains peptidic fragments from caseins. The caseins are amphiphilic phosphoproteins and their isoelectric point (p*I*) value is 4.6. At pH above the p*I*, caseins are negatively charged and soluble in water. The caseins have an electronegative domain preferentially located in small peptidic fragments known as α_s1_-Casein, β-casein and κ-casein [39]. These structural features of the caseins may render these molecules adept at forming complexes with multivalent cationic macromolecules, such as chitosan [38]. In yoghurt (pH = 4.4–4.6) aggregation of the casein-peptide-fragments occur because of a reduction in the electrostatic repulsion at around their pI value.

Anal *et al*. [38] studied the interactions between sodium caseinate and chitosan, under a range of conditions. This study showed that soluble or insoluble chitosan–caseinate complexes can be formed depending on the pH. The characteristics of the complexes are determined by the biopolymer types and their concentration, as well as by environmental conditions. In a certain pH range (5.0–6.0), nanocomplexes of chitosan and sodium caseinate with diameter between 250 and 350 nm were formed. The chitosan and sodium caseinate complexes associated to form larger particles, which resulted in phase separation appear when the pH was either in the range 4.0–4.5 or >6.5. At pH 3.0–3.8, where chitosan and sodium caseinate have similar charges, they may dissociate from each other and become solubilized in solution.

**Figure 3:**
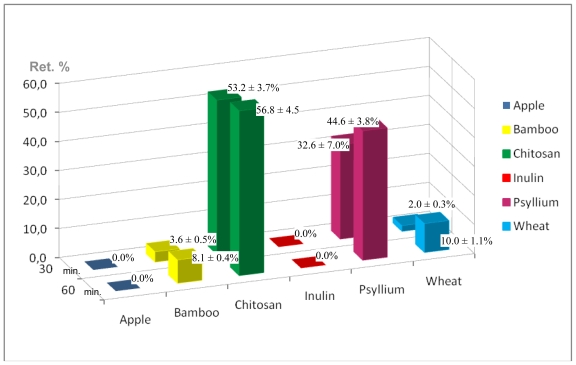
Retention percentages of yoghurts with different studied fibers.

According to these authors, yoghurts with chitosan could contain chitosan-casein-peptidic complexes apart from free chitosan molecules in solution. Besides, we add iron which could interact with free chitosan molecules and those complexes. In our work, yoghurt with chitosan and iron is subjected to the gastrointestinal simulation. In the first step, our food passes through the simulated stomach (pH = 1.0–2.0) and it could be expected that caseins peptidic fragments, chitosan and iron all remain in solution. Changes in pH, while the food passes through the first portion of the simulated small intestine, can lead to formation of chitosan-casein peptidic complexes and iron could be interacting with them. At pH 6.8–7.0, free chitosan molecules and chitosan-casein-peptidic complexes precipitate forming flocculus. The force of the coagulum formed is high and can be seen in Figures 1 and 2. The results reported by Ausar *et al*. [39] indicate that hydrophobicity of the casein-chitosan complex is the main mechanism by which the casein-chitosan flocculation is produced. Iron retention percentages of different fibers are presented in Figure 3. Bamboo and wheat fibers, both insoluble, have low iron retention percentages between 2–5% at 30 min with a maximum of 10% at 60 min. There are no significant differences (p < 0.05) between them by Tukey’s test.

Bamboo and wheat are high in cellulose content. Cellulose could retain iron by physical adsorption according to results reported by Torre *et al*. [13]. They worked with high dietary fiber food materials studying the physicochemical interactions with Fe(II), Fe(III) and Ca(II) without an *in vitro* digestive model. They found that the interaction between Fe(II) and cellulose could be explained better by physical adsorption than complex formation.

Inulin, a soluble fiber, has no iron retention in either assay. This result is in accordance with studies that confirm that inulin does not interfere with iron absorption [20,40–42].

Although psyllium and apple fiber contain both soluble and insoluble fractions, they have significantly different responses (p < 0.05). The apple fiber incorporated in yoghurt has no influence on iron retention. Psyllium shows, on average, 44.6 ± 3.8% iron retention at 60min, which may be mainly attributed to the formation of high viscous dispersion that could be interfering with iron absorption (Figure 2). In addition, the differing behaviors between apple fiber and psyllium could be explained by the different chemical composition of these fibers. Psyllium has high hemicellulose content and apple has the highest lignin content and cellulose. However, bamboo has a low iron retention percentage although its hemicellulose content, (40.2 ± 1.7), is probably because it has cellulose (45.2 ± 1.0) and lignin (5.0 ± 0.3).

Chitosan presents the highest iron retention percentages at 30 min (53.2 ± 3.7%) and 60 min (56.8 ± 4.5%), which shows significant differences (p < 0.05) with other fibers. This biopolymer, which has an animal origin, contains 98% insoluble fiber, and flocculates in the first portion of the small intestine. These flocculus (Figure 2), which could entrap iron, clearly decrease iron dialysis. However, certain amount of iron could go through the cellulose membrane and could be measured to calculate the iron retention percentage. Certain amount of casein-peptide-fragments interacting with iron could remain in solution. Nevertheless, their presence does not interfere with the calculation of iron retention percentages as proven by the digestive simulations performed with control yoghurts. Chitosan is essentially a positively charged polysaccharide and iron is a cation. Anal *et al*. [38] measured zeta potential of chitosan solutions, sodium caseinate solutions and chitosan-caseinate mixtures in a range of pH (3.0–6.5). They found that the pure chitosan solutions were strongly positively charged between pH 3.0 and 6.0. The zeta potential values of chitosan solutions decreased with increasing pH and were slightly negative (approximately −2.5 mV) at pH 6.5. In our study, in this range of pH (3.0–6.0), isolated molecules of chitosan were probably interacting with iron by adsorption rather than by electrostatic forces. Besides, Anal *et al*. [38] found that the zeta potentials of the chitosan–caseinate solutions were negative at pH > 5.5. In this range of pH, in our work, electrostatic interaction could exist between chitosan-caseinate complexes and iron. However, when chitosan precipitates, it captures the iron whether iron interacts with chitosan by electrostatic forces or by adsorption.

This study shows that the effect of chitosan on iron absorption is more pronounced and higher than those measured for the other studied plant fibers, as dietary fiber is a significant factor that influences iron absorption. In the same way, we observed with an *in vitro* study, that when chitosan is added to a food like yoghurt, glucose and calcium availabilities are decreased and this effect is more pronounced than with the other fibers [24]. The iron retention percentages of different fibers used in this work could be explained mainly as a result of physicochemical phenomena, like adsorption, formation of viscous dispersion and flocculus.

*In vitro* methods cannot be used alone for important decisions taken by industry or international organizations because human studies are required for such determinations [43]. The findings presented in this study may be used to increase the understanding of the interactions between chitosan and plant fibers with minerals like iron, for screening purposes.

## 3. Experimental Section

### 3.1. Chitosan

Chitosan was obtained from crustacean chitin in the Laboratorio de Investigación Básica y Aplicada en Quitina (LIBAQ-INQUISUR-CONICET), Universidad Nacional del Sur, Bahía Blanca, Argentina. Chitin firstly was isolated from shrimp (Pleoticus mülleri) waste by the process that was described in our previous work [25].

Chitosan was prepared directly by heterogeneous deacetylation of chitin with 50% (w/w) NaOH. For the biopolymer characterization, moisture and ash contents were determined at 100–105 °C and 500–505 °C, respectively. Deacetylation degree was obtained using FT-IR spectroscopy (Nicolet iS10 FT-IR Spectrometer, Thermo Fisher Scientific, USA) with samples in the form of KBr at a ratio of 1:2. Viscosity of 1% chitosan in 1% acetic acid solution was measured with a Brookfield model DV-IV + viscosimeter (Brookfield, USA) with spindle 21 and a 50 rpm rotational speed at 25 °C.

### 3.2. Plant Fibers

The fibers used were: inulin (Frutafit-inulin, Imperial Sensus, The Netherlands), bamboo (Qualicel, CFF, Gehren, Germany), wheat (Vitacel WF 101, JRS, Rosenberg, Germany), apple (Vitacel AF 400-30, JRS, Rosenberg, Germany) and psyllium (Metamucil, Procter and Gamble Co., Cincinnati, OH, USA). Metamucil is a pharmaceutical formula with Plantago ovata seed husk (49.15% w/w) and sucrose (50.85%). Suppliers of wheat and apple fiber indicated that these products are free from phytic acid, and besides, the wheat fiber is gluten free. The inulin utilized in this work has a degree of polymerization ≥ 9 as declared by suppliers.

### 3.3. Analysis for Dietary Fiber

Total, soluble and insoluble dietary fiber contents of chitosan and plant fibers were analyzed according to the enzymatic–gravimetric method of the Association of Official Analytical Chemists (AOAC) Official Method 991. 43 [27].

Apple, bamboo, psyllium and wheat fibers were investigated to obtain contents of main cell wall constituents (lignin, cellulose, hemicellulose). These components were determined by modifications of the method described by Robertson and van Soest [31] using ANKOM200/220 Fiber Analyzer (ANKOM Technology, Macedon, NY, USA). This method measures Acid Detergent Fiber (ADF), Neutral Detergent Fiber (NDF) and Lignin. Cellulose and hemicellulose contents were obtained by calculations. To determine ADF, duplicate samples were agitated under pressure with hot acid detergent solution for 60 min, rinsed in hot water and dried. To determine lignin content, duplicated samples were digested in 72% (v/v) sulfuric acid, following ADF analysis. Cellulose content of samples was calculated from ADF minus the lignin content. To determine NDF, duplicated samples were shaken with neutral detergent solution and heat-stable α-amylase for 60 min, rinsed and dried. Hemicellulose content of samples was calculated as NDF minus ADF.

### 3.4. Yoghurt Preparation

Yoghurt was prepared using reconstituted whole milk powder with 15% and 5% sucrose. This mix was homogenized and heated to 85 °C for 30 min., cooled to ambient temperature and inoculated with 0.03% starter culture [23]. Starter was constituted by a 1:1 mixture of *Streptococcus thermophilus* (CIDCA collection 321) and *Lactobacillus delbrueckii* subsp. *bulgaricus* (CIDCA collection 332) [44].

Samples were incubated at 43 °C to reach a pH of 4.4–4.6 and stored at 4 °C, after completion of the fermentation process [45]. Samples of yoghurt were added with 1.3% (w/w) of each dietary fiber. The amount of fiber was selected following US regulations for fiber-fortified products [46]. Besides, yoghurts with each type of fiber were added with 0.8% (w/w) of ferrous sulfate. This addition was in accordance with local regulations governing iron supplementation in milk products. Ferrous sulfate (FeSO_4_·7 H_2_O) of 99.9% purity was used as purchased (Sigma-Aldrich Co., St. Louis, MO, USA).

### 3.5. Digestive Chemical Experimental Model

A digestive chemical experimental model was performed to study the interaction of dietary fiber with iron, obtaining iron retention percentages. Experiments were carried out in the following manner.

Yoghurts with ferrous sulfate and each fiber were stirred in 50 mL of 0.1 M HCl (Merck) for 1 h at pH 1.0–2.0, 30 rpm and 37 °C to reproduce the gastric environment. PH was checked each 15 min with a pH Meter Hach model EC-30 (USA) during this first step of simulation and it remained constant (pH 1.0–2.0).

The pH level of these mixes was then increased to pH 6.8–7.2 with 0.2 M NaHCO_3_ (Sigma-Aldrich Co., St. Louis, MO, USA) to reproduce the chemical duodenal environment. The stirring speed was increased from 30 to 300 rpm to imitate the peristaltic movement and temperature was maintained at 37 °C. After 3 min to addition of NaHCO_3_, pH was measured (pH 2.5–3.5) and mixes were immediately transferred into a dialysis tubing cellulose membrane (D9527-100 FT, (Sigma-Aldrich Co., St. Louis, MO, USA). This cellulose membrane (molecular weight cut-off 12,400) was previously prepared, as indicated by suppliers, and it was cut into 28 cm length pieces. The loaded tubes were immersed in 100 mL of distilled water; at 37 °C. Iron concentrations were determined from the dialyzed medium at 30 and 60 minutes. A control yoghurt with ferrous sulfate without fibers was subjected to the digestive simulation and was considered as 0% iron retention (100% iron dialyzated) to calculate iron retention percentages for each fiber.

### 3.6. Iron Content Determination

To determine iron concentration in the dialyzates a spectrophotometric method was used. Iron was reduced with mercaptoacetic acid (succinic acid buffer, pH 3.7). Then, it reacted with piridil bis-fenil trazine sulfonate (PBTS) producing a pink color due to the complex formed (Wiener Lab Fe-colour Kit, Rosario, Argentina). Two mL of reducing medium was mixed with 500 μL of dializated and the absorbance was read on a spectrophotometer (Spectronic 20 Genesys, Thermo Electron Scientific Instruments Corp., Madison, WI, USA) at 560 nm (internal blank). Immediately, one drop of PBTS was added, the solution was mixed, and absorbance was read again after 5 min. All glassware used in sample preparation and analysis was rinsed with 10% (v/v) concentrated HCl (37%) and deionized water before using, to avoid mineral contamination. A regression equation (y = 2.5333x + 0.0042, R^2^ = 0.995) derived from data generated from standards of Fe_2_SO_4_ was used to calculate iron concentrations in the samples.

### 3.7. Iron Retention Percentage Calculations

Iron retention percentages for each studied fibers were calculated as a percentage of the amount of iron measured in the dialyzed medium obtained with the control yoghurt without fibers.

### 3.8. Statistical Analysis

Experiments were performed at least nine times for each dietary fiber using freshly prepared yoghurt. For total iron concentration in dialyzates, each individual sample was run in duplicate. Averages and standard deviations were calculated and expressed in each case as the mean ± SD for n replicates. Normality of the data was checked with the Lilliefors test. The influence of different dietary fibers on the iron retention percentage was statistically analyzed by a one-way analysis of variance (ANOVA) to find significant differences (p < 0.05) and Tukey’s test was used to compare means.

## 4. Conclusions

This study showed that chitosan had the highest iron retention percentage when it was added to a food like iron-fortified yoghurt, as determined by an *in vitro* digestive chemical model. The iron retention percentages of the different fibers used in this work could be explained mainly as a result of physicochemical phenomena, like adsorption, formation of viscous dispersion and formation of flocculus. Chitosan interacted in a more pronounced manner with iron than the plant fibers evaluated under the same conditions.

## Figures and Tables

**Table 1:** Characterization of fibers: Total, soluble and insoluble fiber content (g/100g) according to the enzymatic–gravimetric method of the Association of Official Analytical Chemists (AOAC) Official Method 991.43 [27].

Fiber	Insoluble fiber (g/100g)	% Insoluble fiber	Soluble fiber (g/100g)	% Soluble fiber	Total fiber
Apple	44.8 ± 0.4	77.1	13.3 ± 0.7	22.9	58.1 ± 1.0
Bamboo	91.4 ± 0.5	95.9	3.2 ± 0.8	3.4	95.3 ± 0.9
Chitosan	98.0 ± 1.0	100	nd	nd	98.0 ± 1.0
Inulin	nd	nd	≥85.5	100	≥85.5
Psyllium	37.5 ± 0.6	82.9	7.1 ± 0.5	15.7	45.2 ± 0.8
Wheat	92.1 ± 0.6	97.6	2.3 ± 0.6	2.4	94.4 ± 1.1

**Table 2:** Plant fiber contents of main cell wall constituents (lignin, cellulose, hemicellulose) determined by a modification of the method described by Robertson and Van Soest [31] using ANKOM200/220 Fiber Analyzer.

Fiber	ADF	NDF	Lignin	Cellulose	Hemicellulose
Apple	38.6 ± 0.9	44.3 ± 0.7	8.4 ± 0.8	30.2 ± 1.7	5.7 ± 1.6
Bamboo	50.2 ± 0.7	90.4 ± 0.6	5.0 ± 0.3	45.2 ± 1.0	40.2 ± 1.7
Psyllium	7.3 ± 0.4	36.8 ± 0.9	0.8 ± 0.1	6.5 ± 0.4	29.5 ± 1.3
Wheat	74.8 ± 0.3	89.7 ± 0.6	2.6 ± 0.4	72.2 ± 0.7	14.9 ± 0.9

ADF: Acid Detergent Fiber; NDF: Neutral Detergent Fiber.
